# The Effects of Salt on Rheological Properties of Asphalt after Long-Term Aging

**DOI:** 10.1155/2013/921090

**Published:** 2013-12-25

**Authors:** Xin Yu, Ying Wang, Yilin Luo, Long Yin

**Affiliations:** College of Civil and Transportation Engineering, Hohai University, Nanjing 210098, China

## Abstract

Limited studies in recent years have shown that asphalt pavement subject to seawater in coastal regions or deicing salt in cold regions may be seriously damaged after being soaked in saline water for a long time. However, there is limited research into the influence of salt on rheological properties of asphalt after long-term aging. In this study, rheological properties of unmodified and polymer-modified asphalt after long-term aging were tested after being soaked in different concentrations of salt (0.3%~5%) for different durations (1 day~30 days). Orthogonal array based on the Taguchi method was used for experimental design. The frequency sweep tests were performed on the specimens of aged asphalt after being soaked for complex modulus and phase angle master curves and ultimate fatigue temperature. BBR tests were performed for stiffness. The test results indicate that saline water appears to reduce low temperature properties and fatigue resistance properties and improved high temperature properties of aged asphalt, and it also affects the sensitivity of complex modulus and phase angles at low frequencies.

## 1. Introduction

In some coastal regions, seawater can easily invade asphalt pavement structure under the effect of tide and hurricane and salt will be left on the surface of pavement after evaporation. 77.75% of seawater salt is sodium chloride [1]. The salt accumulated on the surface of asphalt pavement can cause damages to asphalt material due to erosion and crystal formation after dehydration [2]. Meanwhile, a large number of municipal, states, and provincial transportation agencies rely on deicing salt to assure traffic of wintertime mobility and security, especially in the United States, Canada, Japan, and China [3, 4]. Sodium chloride is the world's most commonly used deicing product [5, 6]. Deicing salt with snow invaded into asphalt concrete will accelerate freeze-thaw damage and cause destruction of asphalt pavement [7, 8].

Currently, research on the effects of salt on asphalt and asphalt mixture performance is limited. Some of the existing studies are summarized as follows. Yi-Sha [8] took the soil with Na_2_SO_4_ and NaCl, for example, which generally analyzed effects of salt on penetration, intenerating point, and ductility and then concluded that the penetration and ductility decreased with the increase of concentration of salt, while softening point increased. It was found that the presence of salt will greatly deteriorate the rheological properties of asphalt and the service performance and durability of asphalt will be influenced. Feng et al. [9] studied the impact of salt and freeze-thaw cycles on the performance of asphalt mixtures in the coastal regions of China as well as the effect of salt on the performance of asphalt binders. The research results indicated that the penetration and softening point of virgin asphalt before or after PAV are less influenced by the addition of granule salt. However, salt has a great effect on ductility at 15°C, which indicates the deformability of binders at low temperature. It is also found that when the salt content is more than 3%, the rheological performance of the binder declines sharply. Hassan et al. [10] studied the effects of various antifreeze agents (including various chloride salts antifreeze agent) and found that they have negative impacts on the aggregate and the asphalt mixture in the case of freezing and thawing. They found that freezing and thawing asphalt mixtures in different antifreeze solution causes more damage than freezing and thawing them in pure water. Sinyor et al. [11] performed indirect tensile tests with non-modified asphalt and nanomodified asphalt mixtures after freeze-thaw treatment in NaCl solution. Although the main purpose of the test was to verify that nanomodified asphalt plays a positive role in eliminating cryoprotectants to damage asphalt mixtures, the results also revealed that chemical composition of chlorine salt can greatly affect the strength of asphalt mixtures.

It can be seen that existing research on this topic mostly focused on the impact of salt on the performance of asphalt mixtures, and only one was conducted on asphalt binder using three basic indicators. There is limited research, if any, into the influence of salt on the rheological properties of asphalt binders. Hence, it is necessary to study the effect of salt on rheological properties of aged asphalt. In this study, non-modified asphalt and SBS modified asphalt after long-term aging were soaked in different concentrations of salt at various durations and were subsequently tested for rheological properties.

## 2. Material Preparation and Research Method

### 2.1. Material Preparation

#### 2.1.1. Materials

The experiment used non-modified asphalt (Grade no. 70) and SBS modified asphalt produced in the Jiangsu Province in China.  Table 1 summarizes the various properties of the asphalt binder used in this study, tested in accordance with ASTM.

#### 2.1.2. Preparation of Samples

In coastal regions, asphalt pavements may be soaked in saline water for a long time due to flood inundation caused by high tide, while, in cold regions, soaking in saline water may take place when deicing agents are used in winter. To simulate all possible actual conditions, in this study, different types of asphalt were soaked in water with different salt concentrations and soaking periods. The types of asphalt include conventional bitumen (no. 70 in China) and SBS modified asphalt. NaCl was elected as the salt for conditioning, whose concentrations are chosen to be 0.3%, 1%, 3%, and 5%. Soaking durations are 1 d, 7 d, 15 d, and 30 d. To make the sample preparation and testing more efficient, an orthogonal array via the Taguchi method was used. The orthogonal array used in this study is shown in Table 2.

Before being subjected to soaking, 500 g asphalt was made into specimens of similar shape and thickness. Sixteen groups of specimens in Table 2 were soaked in saline water at 25°C and conditioned at predetermined time. After conditioning, the specimens were taken out and blow-dried by an electrical fan for testing test.

### 2.2. Testing Program

#### 2.2.1. Pressure Aging Vessel (PAV) Test

The short-term aging of the asphalt specimens was achieved by the rolling thin-film oven (RTFO), while the long-term aging was achieved by pressure aging vessel (PAV). The short-term aging test simulates the process of aging in the storage, transport, mixing, and paving. A large number of studies have shown that asphalt aging after PAV is equivalent to the surface layer of asphalt pavement used for 5 years [12, 13]. Dried specimens were aged by using RTFO at 163°C for 85 mins and by using PAV at 90°C and 2.1 MPa for 20 hours to simulate the short-term and long-term aging, respectively.

#### 2.2.2. Bending-Beam Rheometer (BBR) Test

The stiffness (*S*-value) and *m*-value from BBR tests were used to assess the low temperature behaviors of the binders. The low temperature performance of the binders was assessed by the BBR tests at different temperatures (−12°C, −18°C, and −24°C). The *m*-value corresponds to the slope of stiffness (MPa) on logarithmic scale versus time (seconds) at 60 seconds in Superpave design system [14].

#### 2.2.3. Dynamic Shear Rheometer (DSR) Test

DSR of model ATAAR1500^EX^ was used to perform frequency sweep tests for different binders aged in PAV at moderate temperatures (5–80°C). It can generate the complex modulus and phase angle master curves and fatigue factor (G* · sin⁡*δ*). The test conditions are as follows.

- Temperature: 5–80°C (5°C intervals).
- Frequency: 0.1–10 Hz.
- Geometry: 8 mm diameter with 2 mm gap (5–50°C) and 25 mm diameter with 1 mm gap (25–80°C).
- Strain control: 1%.

### 2.3. Flow Chart of the Experiment Procedures

**Figure 1:**
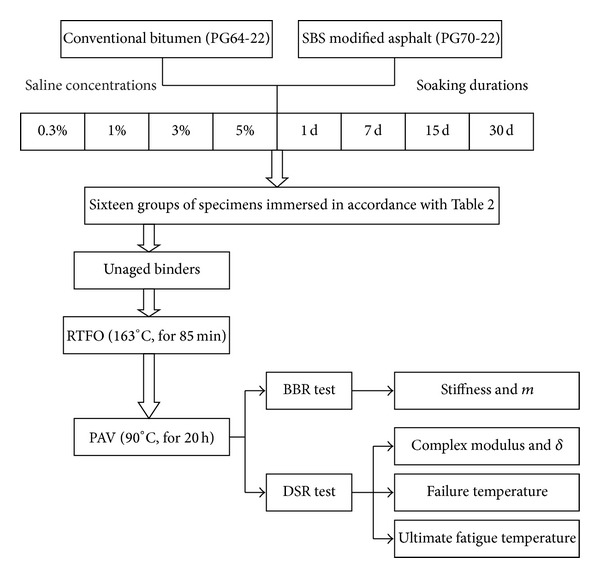
Flow chart of the experimental program.

The flow chart of the experimental procedure is shown in Figure 1.

## 3. Results and Discussions

### 3.1. Complex Modulus and Phase Angle Master Curves

**Figure 2:**
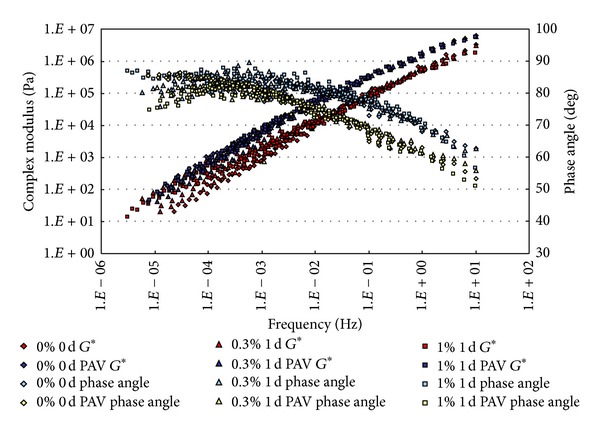
G* and *δ* versus frequency master curves of conventional asphalt soaked in saline water for 1 d.

The conditioned binders, after being aged in RTFO and PAV, were tested in DSR. The strain control for the DSR tests in this study was set as 1%. Based on time-temperature superposition principle (TTSP), the complex modulus (G*) and phase angle (*δ*) master curves (with a reference temperature of 25°C) of these binders were constructed based on the results of frequency sweep tests at temperatures ranging from 25°C to 80°C (5°C intervals). The master curves based on the test results are shown from Figure 2 to Figure 9.

**Figure 3:**
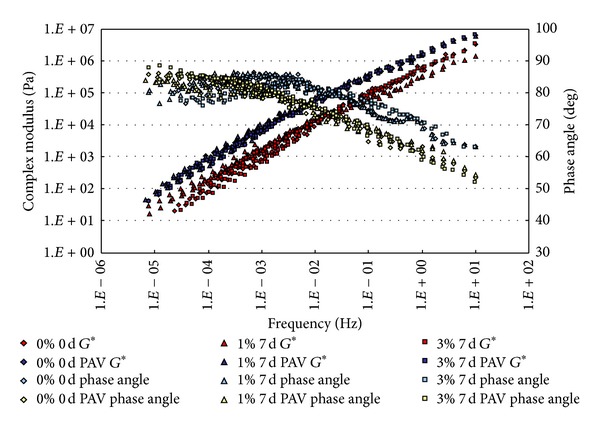
G* and *δ* versus frequency master curves of conventional asphalt soaked in saline water for 7 d.

**Figure 4:**
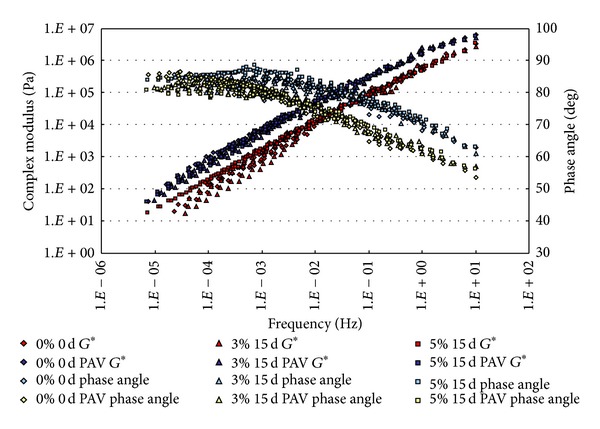
G* and *δ* versus frequency master curves of conventional asphalt soaked in saline water for 15 d.

**Figure 5:**
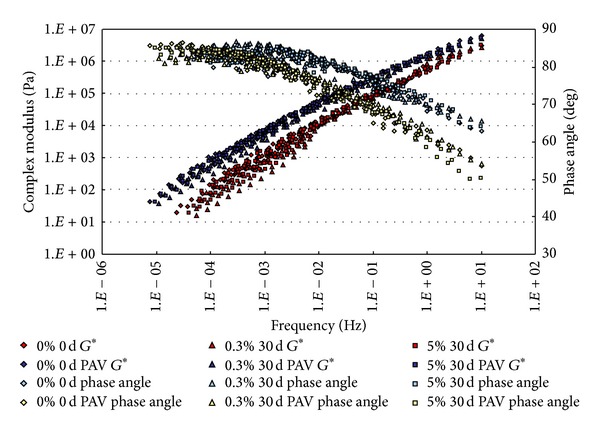
G* and *δ* versus frequency master curves of conventional asphalt soaked in saline water for 30 d.

From Figures 2, 3, 4, and 5, it can be clearly seen that aging after RTFO and PAV increases the complex shear modulus G* and decreases phase angle *δ*. It also appears that soaking in saline water affects the properties of the unmodified, unaged binders. After one day of soaking, there is no large difference between the soaked and unsoaked, unaged specimens. As the soaking period increases, it appears that there are some significant drops in G* at lower frequencies, but there is no difference in *δ*. For aged binders, it appears that there are no differences in G* and *δ* between soaked and unsoaked specimens except for 30 days of soaking, which makes G* slightly drop at low frequency and *δ* decrease at high frequency.

**Figure 6:**
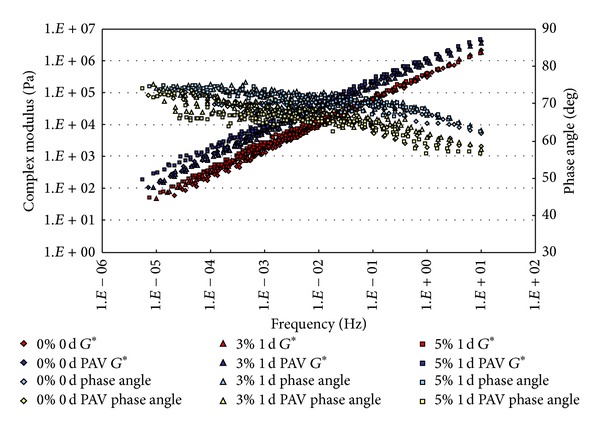
G* and *δ* versus frequency master curves of SBS modified asphalt soaked in saline water for 1 d.

**Figure 7:**
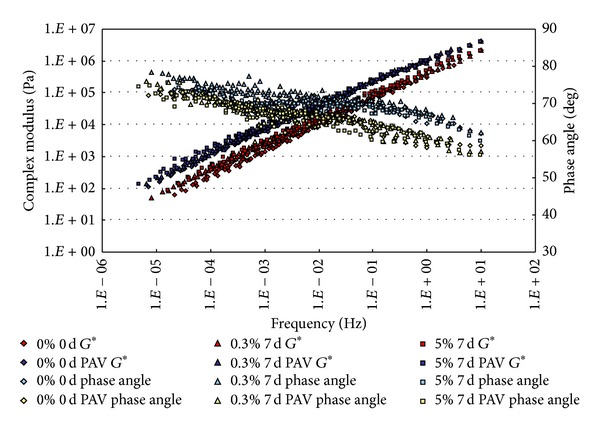
G* and *δ* versus frequency master curves of SBS modified asphalt soaked in saline water for 7 d.

**Figure 8:**
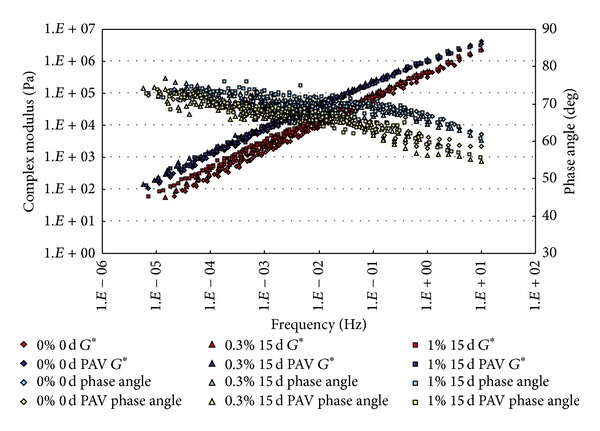
G* and *δ* versus frequency master curves of SBS modified asphalt soaked in saline water for 15 d.

**Figure 9:**
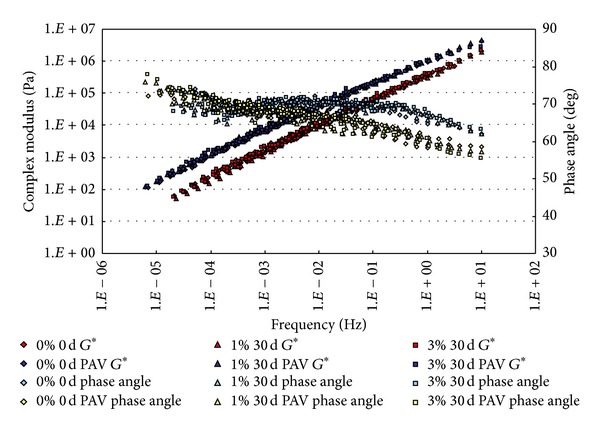
G* and *δ* versus frequency master curves of SBS modified asphalt soaked in saline water for 30 d.

Figures 6, 7, 8, and 9 illustrate that there are no large differences in complex modulus G* and phase angle *δ* for the modified binders soaked in different periods of time, either aged or nonaged. It appears that the polymer-modified binders are less affected by soaking.

### 3.2. Failure Temperature

Various prepared binders were aged in RTFO and PAV and then tested in DSR. The rutting factor, G*/sin⁡*δ*, was measured by every 5°C from 25°C to 80°C. The logarithm of the rutting factor against the temperature is shown in Figures 3 and 4, respectively. Also shown in the figures are the statistically linear regression models developed for the different binders.

**Figure 10:**
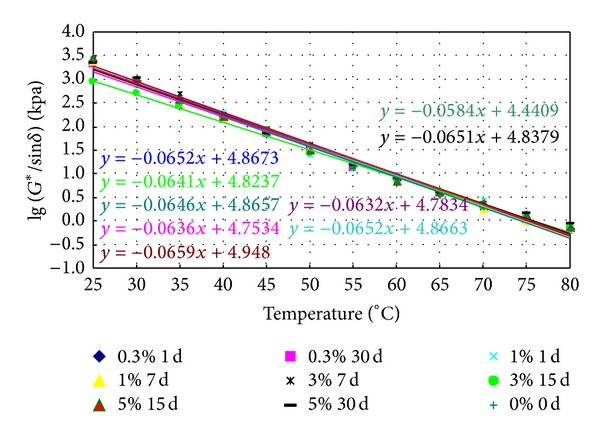
The logarithm of the rutting factor of aged conventional bitumen soaked in saline water.

**Figure 11:**
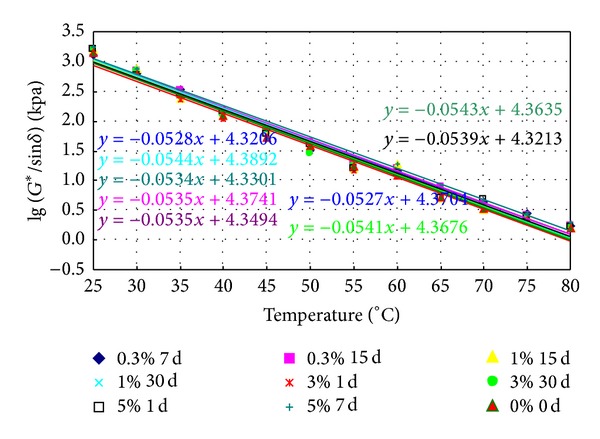
The logarithm of the rutting factor of aged SBS modified asphalt soaked in saline water.

The failure temperature of the rutting factor at 2.2 kPa was obtained by following the linear regression models shown in Figures 10 and 11, and the results are listed in Tables 3 and 4.

From Table 3, it is shown that the values of failure temperature of aged conventional bitumen soaked in saline water increase slightly, which indicates that high temperature properties can be improved. The failure temperature of conventional bitumen soaked in 3% concentrations of salt for 15 days has an increase of 2.18%. When soaking time is fixed, the failure temperature of aged conventional bitumen soaked in saline water first decreases then increases with the increase of saline concentrations. When saline concentration is fixed, the failure temperature of aged conventional bitumen soaked in saline water increases with time.

Table 4 indicates that the values of failure temperature of aged SBS modified asphalt soaked in saline water increase slightly, which means that salt can improve high temperature performance of SBS modified asphalt. The failure temperature of SBS modified asphalt soaked in 5% concentrations of salt for 7 days has an increase of 3.34%. When saline concentration is fixed, the fail temperature of aged SBS modified asphalt soaked in saline water first decreases then increases with time. When soaking time is fixed, the failure temperature of aged SBS modified asphalt soaked in saline water decreases along with the increase of saline concentration.

### 3.3. Creep Stiffness and the *m*-Value

Various binders aged in RTFO and PAV were also assessed by the BBR tests at different temperatures (−12°C, −18°C, and −24°C) to test low temperature performance. Test results of creep stiffness and creep rate (*m*-value) are listed in Tables 5 and 6, respectively.

Table 5 indicates that stiffness of conventional bitumen soaked in saline water after PAV increases at temperature −12°C. When soaked in 3% concentrations of salt for 15 days, stiffness increases by 71.30% at temperature −12°C. Therefore, low temperature properties of the specimen are significantly decreased.

From Table 6, it can be noted that the stiffness of the aged SBS modified asphalt soaked in different saline concentrations for different days increases, especially when soaked in 5% concentrations for 7 days. Increase of *S*-value may reduce its low temperature properties.

### 3.4. Ultimate Fatigue Temperature

**Figure 12:**
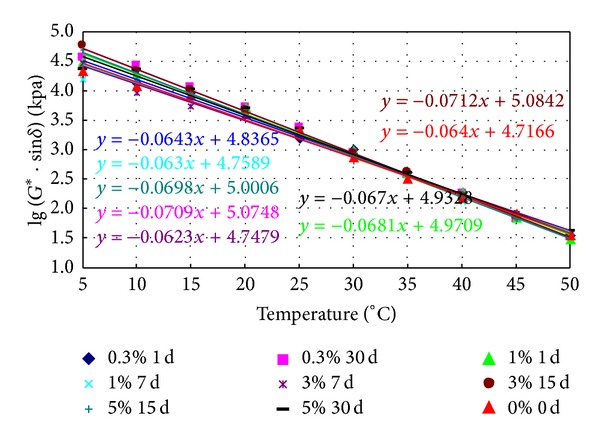
The logarithm of the fatigue factor of aged conventional asphalt soaked in saline water.

**Figure 13:**
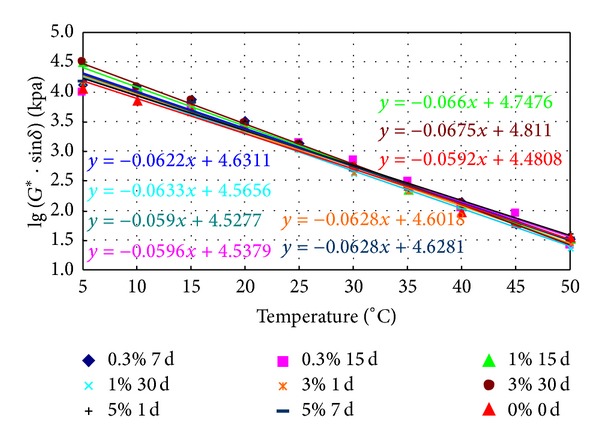
The logarithm of the fatigue factor of aged SBS modified asphalt soaked in saline water.

Various prepared binders were aged in RTFO and PAV and then tested in DSR. The obtained fatigue factor (G* · sin⁡*δ*), commonly believed to reflect the fatigue properties of the binders, was measured from 5°C to 50°C (5°C intervals). The logarithms of the fatigue factor against temperatures are plotted in Figures 12 and 13, which also show the linear regression models relating the two parameters.

It is specified that the G* · sin⁡*δ* of the binder after PAV should be less than 5 MPa at 25°C. We can observe that the logarithm of the fatigue factor decreases proportionally with temperature from Figures 12 and 13; therefore, the lower the temperature corresponds to the 5 MPa in Figures 12 and 13, the “safer” it is for the binder to meet the specification requirement. Hence, the threshold temperature corresponding to 5.0 MPa of the fatigue factor was obtained by using the regression equations and is listed in Tables 7 and 8.

Table 7 shows that the fatigue temperature of aged conventional bitumen soaked in salt increases, which suggests that salt reduces fatigue resistance properties. The fatigue temperature after PAV of conventional bitumen soaked in 3% concentrations of salt for 15 days is the maximum, indicating a maximum reduction in fatigue resistance properties. When soaking time is fixed, the fatigue temperature of aged conventional bitumen soaked in salt first increases then decreases with the increase of saline concentrations.

From Table 8, it can be observed that the fatigue temperature of aged SBS modified asphalt soaked in salt increases, which suggests salt reduced fatigue resistance properties. The fatigue temperature of SBS modified asphalt soaked in 3% concentrations of salt for 30 days after PAV is the maximum, indicating a maximum reduction in fatigue resistance. When the soaking time is fixed, the fatigue temperature of aged SBS modified asphalt soaked in salt first decreases then increases with the increase of saline concentrations. While saline concentrations are fixed, the fatigue temperature of aged SBS modified asphalt soaked in salt firstly decreases then increases with time.

## 4. Conclusions

Using orthogonal experimental design influence of saline water on rheological properties of conventional bitumen and SBS modified asphalt after long-term aging was studied. The asphalt specimens after PAV were tested in BBR and DSR tests for complex modulus and phase angle master curves, stiffness, and ultimate fatigue temperature. Based on the test results, some conclusions can be drawn as follows.

When soaking time is fixed, for aged conventional bitumen, there are no differences in G* and *δ* between soaked and unsoaked specimens except for 30 days of soaking, which makes G* slightly drop at low frequency and *δ* decrease at high frequency. And for SBS modified asphalt after long-term aging, there are also no differences in G* and *δ* between soaked and unsoaked specimens.

At high temperatures, salt can improve appreciably high temperature performance of aged asphalt. When soaking time is fixed, the failure temperature of aged conventional bitumen soaked in saline water first decreases then increases with the increase of saline concentrations; nevertheless, thatof aged SBS modified asphalt soaked in saline water decreases. When saline concentrations are fixed, the failure temperature of aged conventional bitumen soaked in saline water increases with time, but that of aged SBS modified asphalt soaked in saline water first decreases then increases.

Compared to aged conventional bitumen, low temperature properties of the specimens soaked in salt after PAV are significantly decreased, especially when soaked in 3% concentrations of saline water for 15 days. For aged SBS modified asphalt, salt reduces the low temperature performance.

Salt can also reduce fatigue cracking properties of asphalt after long-term aging, evaluated by the fatigue factor (G* · sin⁡*δ*). When soaking time is fixed, the fatigue temperature of aged conventional bitumen soaked in saline water first increases then decreases with the increase of saline concentrations. However, when soaking time is fixed, the fatigue temperature of aged SBS modified asphalt soaked in salt first decreases then increases with the increase of saline concentrations. When salt concentration is fixed, the fatigue temperature of aged SBS modified asphalt soaked in saline water first decreases then increases with time.

Based on the test results, we can conclude that salt has obviously effect on the rheological properties of unmodified asphalt and SBS modified asphalt after PAV, especially for low temperature performance.

## Figures and Tables

**Table 1:** Empirical properties of asphalt.

Inspection item	Test standards	Types of asphalt	
		Conventional bitumen	SBS modified asphalt
		Test results	Test results
Penetration (25°C, 100 g, 5 s)/0.1 mm	ASTM D 5	65.4	69.5
Ductility (15°C, 5 cm/min)/cm	ASTM D 113	>100	34.6
Softening point (TR & B)/°C	ASTM D 36	47.3	73.7
Density (15°C)/(g/cm^3^)	ASTM D 70	1.034	1.037
Solubility (trichloroethylene)/(%)	ASTM D 2042	99.9	99.6
PG grade	ASTM D 946	PG64-22	PG70-22

**Table 2:** Form of orthogonal experiment.

Test group numbers influence factors	Types of asphalt	Saline concentrations	Soaking durations
1	1	1	1
2	1	2	2
3	1	3	3
4	1	4	4
5	1	1	4
6	1	2	1
7	1	3	2
8	1	4	3
9	2	1	3
10	2	2	4
11	2	3	1
12	2	4	2
13	2	1	2
14	2	2	3
15	2	3	4
16	2	4	1

Types of asphalt ((1) conventional bitumen (no. 70 in China), (2) SBS modified asphalt); saline concentrations ((1) 0.3%, (2) 1%, (3) 3%, and (4) 5%); soaking durations ((1) 1 d, (2) 7 d, (3) 15 d, and (4) 30 d).

**Table 3:** Failure temperature after PAV of conventional bitumen soaked in saline water.

Conventional bitumen	0% 0 d	0.3% 1 d	0.3% 30 d	1% 1 d	1% 7 d	3% 7 d	3% 15 d	5% 15 d	5% 30 d
Failure temperature (°C)	73.10	73.44	73.49	73.42	74.02	73.88	74.69	74.09	74.43

**Table 4:** Failure temperature after PAV of SBS modified asphalt soaked in saline water.

SBS modified asphalt	0% 0 d	0.3% 7 d	0.3% 15 d	1% 15 d	1% 30 d	3% 1 d	3% 30 d	5% 1 d	5% 7 d
Failure temperature (°C)	78.70	80.33	80.28	79.27	79.23	79.82	78.90	79.61	81.43

**Table 5:** Creep stiffness *S*- and *m*-value after PAV of conventional bitumen soaked in saline water.

Conventional bitumen		0% 0 d	0.3% 1 d	0.3% 30 d	1% 1 d	1% 7 d	3% 7 d	3% 15 d	5% 15 d	5% 30 d
−12	*S* (MPa)	108	124	116	178	127	131	185	131	112
	*m*	0.380	0.378	0.374	0.473	0.366	0.374	0.388	0.377	0.376
−18	*S* (MPa)	262	259	295	335	303	268	274	354	282
	*m*	0.307	0.291	0.302	0.297	0.270	0.292	0.294	0.278	0.302
−24	*S* (MPa)	571	728	586	720	612	642	406	665	655
	*m*	0.205	0.213	0.220	0.233	0.196	0.215	0.246	0.203	0.212

**Table 6:** Creep stiffness *S*- and *m*-value after PAV of SBS modified asphalt soaked in saline water.

SBS modified asphalt		0% 0 d	0.3% 7 d	0.3% 15 d	1% 15 d	1% 30 d	3% 1 d	3% 30 d	5% 1 d	5% 7 d
−12	*S* (MPa)	84.3	100	88.3	88.0	90.0	93.6	100	94.9	118
	*m*	0.399	0.375	0.368	0.413	0.377	0.400	0.395	0.399	0.389
−18	*S* (MPa)	197	218	247	204	224	231	193	251	231
	*m*	0.316	0.334	0.329	0.314	0.340	0.317	0.331	0.328	0.335
−24	*S* (MPa)	508	474	473	424	391	492	240	526	587
	*m*	0.230	0.241	0.226	0.210	0.176	0.228	0.219	0.248	0.253

**Table 7:** Fatigue temperature of aged conventional bitumen soaked in salt.

Conventional bitumen	0% 0 d	0.3% 1 d	0.3% 30 d	1% 1 d	1% 7 d	3% 7 d	3% 15 d	5% 15 d	5% 30 d
FT_*f*_	15.9	17.7	19.4	18.7	16.8	16.3	19.5	18.6	18.4

**Table 8:** Fatigue temperature of aged SBS modified asphalt soaked in salt.

SBS modified asphalt	0% 0 d	0.3% 7 d	0.3% 15 d	1% 15 d	1% 30 d	3% 1 d	3% 30 d	5% 1 d	5% 7 d
FT_*f*_	13.2	15.0	14.1	15.9	13.7	14.4	16.5	14.1	14.8
